# Intra-vagal parathyroid adenoma on digital PET/CT with ^18^F-fluorocholine

**DOI:** 10.1007/s00259-021-05452-7

**Published:** 2021-06-17

**Authors:** Ismini C. Mainta, Marie-Laure Matthey Gié, Frédéric Triponez, Martin A. Walter

**Affiliations:** 1 Division of Nuclear Medicine, Diagnostic Department, University Hospital of Geneva, Rue Gabrielle-Perret-Gentil 4, 1205 Geneva, Switzerland; 2 Division of Thoracic and Endocrine Surgery, Department of Surgery, University Hospital of Geneva, Rue Gabrielle-Perret-Gentil 4, 1205 Geneva, Switzerland

Parathyroid adenoma within or adjacent to the vagus nerve is a rare ectopia, with only 21 cases reported in the literature [1–3]. A plausible explanation described by Gilmour in 1937 is that parathyroid elements, destined for the inferior parathyroid glands, may segregate and attach to the nerve during embryogenesis [4, 5].

We present a 49-year-old woman with primary hyperparathyroidism complicated by osteoporosis (T-score: − 3.1 SD), over 30 episodes of urolithiasis necessitating invasive treatment, and recurrent acute pancreatitis despite the absence of additional risk factors. Ultrasound, technetium-99 m-sestamibi scintigraphy (a), three cervical explorations, and one thoracoscopic thymectomy by experienced surgeons at other medical centers were unsuccessful in revealing a parathyroid adenoma.

Eventually, the patient underwent a fluorine-18-fluorocholine PET/CT with a digital PET scanner (Biograph Vision, Siemens). This scan revealed a 7 mm intra-vagal parathyroid adenoma with high fluorine-18-fluorocholine uptake (maximum intensity projection, b; axial PET/CT, c). The contrast-enhanced CT (d) and the 3D reconstruction of the PET/CT (e) illustrate the unusual location of the parathyroid adenoma between the left internal carotid artery and the jugular vein, 2 cm cranially to the carotid bifurcation. The nodule was successfully resected, and subsequently, the serum parathyroid hormone decreased from 25 to 0.9 pmol/L (reference values: 1.1–6.8 pmol/L).

The improved spatial resolution and contrast of digital PET facilitate the detection of small lesions [6]. Together with the high accuracy of fluorine-18-fluorocholine PET [7], it provides the opportunity to improve the diagnostic work-up and thus the surgical management of patients with intra-vagal parathyroid adenoma.


